# Mitochondrial Mediated Apoptosis Evaluation of Myristicin on Triple Negative Breast Cancer Model Via Bcl2/Bid Pathway

**DOI:** 10.1002/cbf.70280

**Published:** 2026-08-11

**Authors:** Sudhina Sufina Nazar, Aswathy Vadakkumkattle Ajaykumar, Janeesh Plakkal Ayyappan

**Affiliations:** ^1^ Translational Nanomedicine and Lifestyle Disease Research Laboratory, Department of Biochemistry University of Kerala, Kariavattom campus Thiruvananthapuram Kerala India; ^2^ Centre for Advanced Cancer Research, Department of Biochemistry University of Kerala, Kariavattom campus Thiruvananthapuram Kerala India; ^3^ Department of Anatomy P K Das Institute of Medical Sciences, Vaniyamkulam, Ottapalam Palakkad Kerala India

**Keywords:** apoptosis, mitochondrial stress, myristicin, ROS, TNBC

## Abstract

Among 15‐20 cases of breast cancer, triple‐negative breast cancer (TNBC) is the deadliest form of the disease. The most effective form of treatment for this type of cancer is still targeted chemotherapy because it lacks hormone receptors. Myristicin, an active aromatic compound with anticancer properties, is mostly found in nutmeg. Antitumor, antioxidant, and antimicrobial activity are among few of the numerous properties of myristicin. On TNBC cells, the exact modes of action are mostly unidentified. This study shows that myristicin triggered the mitochondria‐mediated apoptosis in MDA‐MB‐231 cells. The MTT assay assessed the anti‐proliferative potential of myristicin on TNBC cells (IC_50_ 0.65 mM ± 0.98). Flow cytometry analysis was used to evaluate the myristicin's effects on cell apoptosis using annexin V/PI (46.4 ± 2.31%). After evaluating the protein expression, myristicin significantly decreased the expression of Bcl2 and HSP60 while enhancing the expression of proteins such as caspase 9, caspase 3, bid, bad, caspase 7, P53, cytochrome c, and SDHA. Furthermore, our research confirmed that myristicin has a lower toxicological profile and greater in vivo therapeutic efficacy. In BALB/c mice, 4T1 cells were injected subcutaneously to develop breast tumors, and the mice subsequently received myristicin. According to in vivo results, myristicin treatment reduced tumor weight and volume when compared to the breast cancer control group. Besides, the enzyme analysis, gene, and protein expression showed significant apoptotic properties on myristicin treated group. According to these results, myristicin may be used as a therapeutic approach for the management of TNBC.

## Summary

The current study provides insight into the molecular mechanism of Myristicin to demonstrate anticancer activity in a TNBC breast cancer model. The Myristicin significantly triggered the cytotoxic effects on MDAMB‐231 cells via the induction of the apoptosis and cell cycle regulation. In vivo results proved that myristicin reduced the tumor weight and volume of the 4T1 cells induced Balb/c breast cancer model compared to the control group. Besides, the enzyme analysis, gene, and protein expression showed significant mitochondrial‐mediated apoptotic mechanisms on myristicin treated group. Finally, our findings suggest that myristicin has the potential to serve as a valuable platform for breast cancer.

**Abbreviations**
ALP: Alkaline Phosphatase
AO/EtBr: Acridine Orange/Ethidium Bromide
BC: Breast cancer Control
CK: Creatine Kinase
DHE: Dihydroethidium
DMSO: dimethyl sulfoxide
FBS: Fetal bovine serum
GST: Glutathione Transferase
LDH: Lactate Dehydrogenase
M: Myristicin
MDA: Malonaldehyde
MTT: 3‐(4,5‐dimethylthiazol‐2‐yl)‐2,5‐diphenyltetrazolium bromide
PC: Positive Control
ROS: Reactive Oxygen Species
RPMI 1640 Medium: Roswell Park Memorial Institute
SGOT: Serum Glutamic Oxaloacetic Transaminase
SOD: Superoxide Dismutase
TM: Tamoxifen

## 1 Introduction

Breast cancer is the most prevalent type of cancer in women worldwide and has an important impact on the incidences of morbidity and mortality [1]. As a diverse disease, breast cancer comes with an extensive variety of biological traits and clinical symptoms. Three receptors ‐ the estrogen receptor (ER), the progesterone receptor (PR), and the human epidermal growth factor receptor‐2 (HER‐2) – distinguish various subtypes of breast cancer. All three receptors are negative in triple‐negative breast cancer. Triple‐negative breast cancer (TNBC) is an invasive histological subtype that has a poor prognosis and a significantly higher recurrence rate was observed following chemotherapy compared to other subtypes [2, 3].

TNBC is distinguished by a poor prognosis, recurrent metastases, and extensive genetic heterogeneity [4, 5, 6]. Patients with TNBC can only be cured through surgery, radiation, and chemotherapy [7]. While a variety of chemotherapy medicines are employed in treating breast cancer, they generally cause adverse effects even if they can either destroy tumor cells or stop their progression. Regretfully, the use of chemotherapy and radiation therapy is restricted by drug resistance, adverse effects, and poor therapeutic outcomes. The primary drawbacks of the widely utilized therapeutic treatments for TNBC are their inability to identify specific molecular targets and the poor sensitivity/specificity of the existing immunohistochemistry biomarkers [8]. The conventional approach of controlling TNBC metastases is to trigger apoptosis. Therefore, strengthening integrated cancer treatment strategies requires the finding and development of innovative anti‐cancer compounds [9].

Many of the anticancer treatments at present in use are ineffective due to their adverse consequences as well as their lack of treatment efficiency. Natural foods, fruits, or plants have gained recognition in the last few years as an essential asset for the creation of innovative and revolutionary cancer treatments [10, 11]. For instance, chemotherapy drugs originating from plants, such as vinorelbine, vincristine, or paclitaxel, have demonstrated efficacy in human cancer research [12, 13, 14]. Dietary components contain phytochemicals that have demonstrated exciting anticancer activities. Myristicin is one of the allylbenzenes found naturally in many spices, food flavors, and some plant groups (such as the Apiaceae, Lamiaceae, Myrtaceae, and Rutaceae) [15]. The key component in the aromatic ether fraction of the mace essential oil is myristicin. The nutmeg aromatic fraction primarily consists of myristicin, elemicin, and safrole, which comprise 85–95% of the compounds, whereas myristicin serves as 4–12% of the essential oil's constituents [16]. The information gathered so far about allylbenzenes has centered primarily on their carcinogenic properties [17, 18, 19]. Therefore, further research on these dietary components is necessary to enhance risk assessment, and additional data on other biological activities is required. Consistent with our prior research, myristicin was shown to promote apoptosis in estrogen receptor–positive MCF‐7 breast cancer cells without exerting harmful effects on normal WRL‐68 human cells [20].

In the last decade, extensive studies were conducted to develop newer therapeutic targets for TNBC, including inhibitors of cell cycle checkpoints, PI3K–Akt–mTOR, MEK pathway, immune checkpoints, and epidermal growth factor receptor (EGFR), vascular endothelial growth factor, androgen receptor pathways, and antibody–drug conjugates. But unfortunately, there has been little clinical success of targeted therapy, making most patients with TNBC still mainly dependent on conventional chemotherapy [12, 13, 14]. In this study, to establish the in vitro and in vivo role of Myristicin on TNBC, the apoptosis induction of Myristicin was investigated via the mitochondrial stress‐mediated mechanisms.

## 2 Materials and Methods

Myristicin, Paclitaxel, Tamoxifen, Hoechst stain, acridine orange, ethidium bromide, Hematoxylin, Eosin, CM‐H2DCFDA (5‐(and‐6)‐chloromethyl‐2′,7′‐dichlorodihydrofluorescein diacetate, acetyl ester), and dimethyl sulfoxide (DMSO) were purchased from Sigma‐Aldrich. Invitrogen™ (United States) supplied the trypsin–EDTA, fetal bovine serum (FBS; Gibco Life Technologies), BCA Protein Assay kit, Gene JET RNA purification kit, Power SYBR® Green Master Mix, and Verso cDNA Synthesis kit and High‐glucose Dulbecco's minimal essential medium (DMEM). Himedia (Maharashtra, India) supplied the MTT (3‐(4, 5‐dimethylthiazol‐2‐yl)‐2,5‐diphenyltetrazolium bromide) assay kit, antibiotic penicillin‐streptomycin‐amphoterricin B, and Propidium Iodide. Lactate Dehydrogenase (LDH), Malonaldehyde (MDA), Catalase, Superoxide dismutase (SOD), Glutathione Transferase (GST), and JC‐1 dye assay kits were purchased from Origin. All forward and reverse real‐time PCR primers were synthesized by APJ Scientific Solutions (Trivandrum, India). All the primary and secondary antibodies were purchased from Cell Signaling Technology Inc. (Massachusetts, USA). All other materials used were analytical grade.

### 2.1 Cell Culture

Human triple‐negative breast cancer cell MDAMB‐231 (CVCL_0062), human normal hepatocyte cell line WRL‐68 (CVCL_0571), and 4T1 (CVCL_0125) murine breast cancer cell lines were purchased from NCCS, Pune, India and ATCC, VA, USA, respectively (Passage No: 22,19 and 15). According to our understanding, all these cells served as well‐established models for breast cancer research and have been extensively utilized in prior studies. Cells were observed for mycoplasma contamination through the Hoechst staining method, and all assays were conducted using mycoplasma‐free cells. Cells were subcultured in DMEM medium containing 10% FBS and 100 units/mL penicillin/streptomycin/amphotericin B under 5% CO_2_ at 37°C in an incubator.

### 2.2 Cytotoxicity Analysis

Myristicin was supplied as a stock solution in dimethyl sulfoxide (DMSO) at a concentration of 50 mg/50 μL and was diluted with complete culture medium to obtain the desired working concentrations. The final concentration of DMSO across all treatment groups was kept at ≤ 0.1% (v/v), and an identical concentration of DMSO was utilized as the vehicle control. Cell toxicity was analyzed by the MTT assay kit (Himedia). MDAMB‐231 cells were cultured in a 96‐well plate and treated with different doses (0.01, 0.05, 0.1, 0.2, 0.5, 1, and 5 mM) of myristicin were administered for 24 h. After removing the media with 10 μL of MTT solution was added at 37°C in the dark. Subsequently added 100 μL of DMSO in each well after the 2–4‐h incubation and the absorbance was measured at 570 nm using a microplate reader The absorbance at 570 nm with a microplate reader (EVOS XL Core).

### 2.3 Morphological Analysis

Phase‐contrast microscopy was used to analyze morphological changes induced by myristicin in order to distinguish between normal and apoptotic cell death mechanisms. MDA‐MB‐231 cells were treated with myristicin at concentrations of 0.5 and 0.75 mM for 24 h after being seeded in 96‐well plates at a density of 1 × 10^4^ cells per well. After treatment, cellular morphology was analyzed using thermo imaging software of a phase‐contrast microscope.

### 2.4 Morphological Detection of Apoptosis by Hoechst Staining

Morphological characteristics of apoptotic cells were determined using Hoechst staining. Briefly, 1 × 10^4^ cells were seeded in 96‐well plates and cultured for 24 h. Cells were treated with various concentrations of myristicin for 8 h and stained with 50 μL of Hoechst solution for 20 min at CO_2_ incubator. Cells were imaged under a fluorescent microscope at an excitation/emission of 360/460 nm (ZOE Fluorescent Cell Imager, Bio‐Rad).

### 2.5 Live‐Dead Assay

Acridine orange and ethidium bromide dual staining is mainly used to identify cell membrane damage. A 96‐well microplate was used to seed the cells (1 × 10^4^ cells/well), and they were subsequently treated for 8 h with varying doses of myristicin. The cells were treated with dual stain, and they were then placed in the dark for 10 min. After the cells were properly washed with PBS, images of the cells were acquired using a fluorescence microscope. (ZOE Fluorescent Cell Imager, Bio‐Rad).

### 2.6 Apoptosis Determination Using Annexin‐V/PI Staining by Flow Cytometry

Annexin‐V/PI staining was used to detect the apoptotic cells. Briefly, cells were seeded at a density of 2 × 10^5^ cells in 6‐well plates and cultured for 24 h to allow cell attachment to occur. Cells were treated with various concentrations of myristicin and then incubated for 24 h. Cells were harvested by trypsinization and centrifuged. The supernatant was discarded, and cell pellets were washed with PBS before being stained with Annexin‐V FITC/PI. The cell pellets were then resuspended in 195 μL of 1× binding buffer and 5 μL of Annexin‐V and incubated at room temperature for 15 min. After washing with buffer, added 10 μL of PI solution with 190 μL of 1× binding buffer, and the samples were immediately analyzed by flow cytometry (Beckman Coulter, Epics XL MCL). Flow cytometric data were analyzed using Flow Jo 10 software. Fluorescence distribution was displayed as a percentage of fluorescent cells in each quadrant.

### 2.7 Assessment of Mitochondrial Membrane Potential

The mitochondrial membrane potential (ΔΨm) was assessed by the JC‐1 Mitochondrial Membrane Potential Kit as per the manufacturer's protocol (Origin, India). Cells were seeded in black 96‐well plates at a density of 1 × 10^4^ cells/well and allowed to incubate for 24 h. The cells were then incubated with various concentrations of myristicin for an additional 24 h. After treatment, the culture medium was carefully removed, and 100 μL freshly prepared JC‐1 staining solution was added to each well. Plates were placed in a CO2 incubator at 37°C for 20 min. The cells were washed with the assay buffer provided to remove excess dye and fluorescence images were captured using a ZOE Fluorescent Cell Imager (Bio‐Rad) to assess changes in mitochondrial membrane potential.

### 2.8 Antimigration Assay

The scratch assay was used to examine the activity of myristicin on cell migration of MDA‐MB‐231 cells. Cells were seeded in six‐well plates until the cells attained 70% confluence. After that, sterile tips were used to produce a wound by scratching the cell layers. Following a PBS wash, the scratched layer was subjected to varying myristicin concentrations and incubated for an additional 24 h. Under the phase‐contrast microscope (ZOE Fluorescent Cell Imager, Bio‐Rad) at 4× magnification, the migration of cells was examined at various intervals.

### 2.9 Measurement of Intracellular ROS Generation

After being treated in triplicate with various concentrations of myristicin, about 70% of confluent MDAMB‐231 cells were incubated for 8 h. Following that, DCFDA stain was transferred to each well at a final concentration of 10 µM, and the wells were incubated for 30 min in the dark. The fluorescence images were analyzed using a fluorescence microscope (ZOE Fluorescent Cell Imager, Bio‐Rad) at 495 nm excitation and 529 nm emission following incubation. The wells include untreated cells that act as controls, myristicin, and paclitaxel. ROS generation was expressed by an increase in fluorescence compared to untreated control cells.

### 2.10 Reverse Transcription Quantitative Polymerase Chain Reaction (qPCR) Analysis

The expression of apoptotic genes was investigated using reverse transcription‐qPCR (BIORAD). To put it briefly, cells were seeded in 6‐well plates at an average density of 1 × 10^5^ cells/well and placed for 24 h at a CO_2_ incubator. According to the manufacturer's instructions, cells were obtained using TRIzol reagent (Origin) for total RNA isolation following treatment with varying concentrations of Myristicin (0.5 mM and 0.75 mM). afterwards the manufacturer's instructions, the Verso cDNA kit (Thermo Fisher Scientific, Inc.) was employed for generating the cDNA from RNA. The SYBR Green master mix (Thermo Fisher Scientific, Inc.) was used to measure the mRNA levels of Caspases8, Bax, Bid, Bcl2, PARP, p53, and cyclin‐dependent kinase 1 (Cdk1), as well as the housekeeping gene GAPDH (the sequences are provided in Table 1). The PCR was performed 40 times for 20 s at 95°C and for 30 s at 60°C after being started for 3 min at 95°C.

**Table 1:** The summary of human and Mice primer sequences was used for RT‐PCR.

Gene	Forward Primer Sequences (5'‐3')	Reverse Primer Sequences (5'‐3')
H‐Bax	TCCAGGATCGAGCAGGGCGAA	CGATGCGCTTGAGACACTCGCT
H‐Caspase 9	CCAGAGATTGCGAAACCAGAGC	GAGCACCGACATCACCAAATTC
H‐Bcl2	GAACTGGGGGAGGATTGTGG	TTCACTTGTGGCCCAGATAGG
H‐P53	TGTGGAGTATTTGGATGACA	GAACATGAGTTTTTTATGGC
H‐Survivin	TGGCCGCTCCTCCCTCAGAAAA	GCTGCTGCCTCCAAAGAAAGCG
H‐PARP	GGCCTCGGTGGATGGAATG	GCAAACTAACCCGGATAGTCTCT
H‐BID	AGCGCAAACTTAGCCTGTCTG	TCCGGGATGGTGAGATCCTTC
H‐Caspase 8	ACACAGTCGAGTAGACTCTCAAA	AGGAAGTGATGCTCGTTCAGA
H‐FAAD	GCTGGCTCGTCAGCTCAAA	ACTGTTGCGTTCTCCTTCTCT
H‐Cdk1	GGGTCAGCTCGCTACTCAAC	AAGTTTTTGACGTGGGATGC
H‐Caspase 3	TGGAAGCGAATCAATGGA	TGTACCAGACCGAGATGTC
H‐GAPDH	TCCTGCACCACCAACTGCTTAG	GGCATGGACTGTGGTCATGAGT
M‐Bcl2	CATGCCAAGAGGGAAACACCAGAA	GTGCTTTGCATTCTTGGATGAGGG
M‐Bax	GGCTGGACACTGGACTTCCT	GGTGAGGACTCCAGCCACAA
M‐Caspase 9	GATGCTGTCCCCTATCAGGA	GGGACTGCAGGTCTTCAGAG
M‐ caspase 8	GGCCTCCATCTATGACCTGA	GCAGAAAGTCTGCCTCATCC
M‐NFKB	ATGGCAGACGATGATCCCTAC	TGTTGACAGTGGTATTTCTGGTG
M‐TLR2	TCTAAAGTCGATGATCCGCGACAT	CTACGGGCAGTGGTGAAAACT
M‐GAPDH	GCTGGTGCTGAGTATGTCGT	AGTCTTCTGGGTGGCAGT

### 2.11 Western Blot Analysis

The Western blot technique was employed to examine the expression of the protein that triggers apoptosis. In short, cell pellets treated with different concentrations of myristicin for 24 h were obtained using RIPA buffer and the estimated total protein. The protein was separated using Bio‐Rad's 10%–15% sodium dodecyl sulfate polyacrylamide gel electrophoresis (SDS–PAGE), subsequently transferred to a nitrocellulose membrane, and blocked for 1 h with 5% skimmed milk. The primary antibodies (Cleaved PARP, Cytochrome C, Caspase 9 & PDI) were incubated at 4°C overnight at a dilution of 1:1000 (CST) following washing with Tris‐buffered saline (TBST). Following this, membranes were washed and placed for 1 h at a 1:2500 dilution with HRP‐conjugated secondary antibody. Protein bands were subsequently formed using enhanced chemiluminescence under the ChemiDoc™ MP System with IMAGE LAB™ Software (Bio‐Rad). As the loading control, rabbit monoclonal anti‐beta actin (abdos) was employed. ImageJ software (NIH, Bethesda, MD, USA) was used to determine the optical density (OD) of response bands.

### 2.12 In Vivo Experiments

Female BALB/c mice weighing 20–25 g and aged six‐8 weeks were provided by the Sree Chitra Tirunal Institute for Medical Sciences and Technology, Trivandrum. Mice were housed in sterile conditions with cycles of light and dark lasting 12 h each. The initial procedure involved injecting 2 × 10^5^ 4T1 cells in 100 µL of RPMI‐1640 subcutaneously into the right flank of each mouse. Once the tumor reached 80 mm^3^ in size, we randomly divided the mice into five groups. Over a period of 3 weeks, the mice received intraperitoneal injections of myristicin 100 mg/kg, tamoxifen 20 mg/kg, or saline (control) every other day. To assess for toxicity, 100 mg/kg of myristicin was also injected into normal mice. For the in vivo study, myristicin was dissolved in dimethyl sulfoxide (DMSO) to prepare a stock solution and then diluted with sterile normal saline (0.9% NaCl) to obtain the required dosing concentration. The final DMSO concentration was maintained below 5% (v/v) to minimize vehicle‐related effects. The Vernier callipers were used to measure the tumor size in two perpendicular dimensions once every 4 days, and the results were equated to tumor volume. The animals’ body weight was recorded once every 4 days concurrently with an assessment of the tumor's dimensions, and the mortality rate was observed every day. Animals' survival outcomes were characterized as the duration until the moribund condition (or death), at which time the mice were euthanized. Survival curves were created employing the Kaplan–Meier technique.

On the 25th day following the treatments, all of the mice were sacrificed, and the tumor was separated and measured. Following their harvest, sections of the major organs: liver, spleen, kidney, heart, and lung were stained with H&E. The equation was used to calculate the tumor's size:

$$V\left(\text{mm3}\right){=\text{LW}}^{2}/2$$

where L and W stand for the tumors’ respective lengths, and V for the volume.

Additionally, the inhibition rate was calculated using the equation below [21]:

Tumorinhibitionrate(TIR,%)=Wc−Wt/Wc×100

The average weight of the tumors in the control and treated groups is denoted by Wc and Wt, respectively. The Ethical Committee for Animal Experimentation authorized the experimental protocol, and all experiments were conducted in compliance with the committee's guidelines for the control and supervision of animal experiments (CPCSEA). The mice were randomly divided into five experimental groups:

Group 1, normal control mice (Saline)

Group 2, normal control mice treated with Myristicin (100 mg/kg)

Group 3, Tumor‐induced mice (Saline).

Group 4, Myristicin treated with 100 mg/kg.

Group 5, Tamoxifen (positive Control) treated with 20 mg/kg.

### 2.13 Antioxidant Enzyme Analysis

Catalase, superoxide dismutase (SOD), and Glutathione transferase (GST) are examples of antioxidant enzymes that provide the body's primary defense against free radicals and ROS. Briefly, cells were exposed to different doses of myristicin for 24 h. According to the manufacturer's instructions, the intracellular enzyme activity in the mice serum was measured using a catalase, SOD, and GST colorimetric test kit (Origin). The results were expressed as U/ml serum.

### 2.14 LDH and Malondialdehyde (MDA) Assay

In accordance with the manufacturer's instructions, the LDH assay kit (Origin) was used to detect the LDH activity in the serum. Ultimately, the assay was conducted using the collected mice serum. In a microplate reader, the absorbance was measured at 450 nm (Epoch Bio Tech).

MDA, a byproduct of lipid peroxidation and a marker of cellular damage and many types of cancer, was measured using an MDA detection kit (origin). The colorimetric examination was used to measure the amount of MDA‐thiobarbituric acid (TBA) adduct that formed when MDA and TBA reacted. In accordance with the manufacturer's instructions, the relative concentration of MDA in the serum was evaluated.

### 2.15 Assessment of In Vivo Toxicity

The collected blood specimens were kept for 2 h at room temperature. Serum was collected following a 15‐min centrifugation at 840 g. As directed by the manufacturer, serum SGOT, ALP, creatine Kinase, and urea were measured using the appropriate kits (Origin, Cayman).

### 2.16 Statistical Analysis

For statistical analyses, GraphPad Prism, version 9 for PC (GraphPad Software, San Diego, CA, USA), was used. The data is displayed as mean ± SD. Every experiment was run at least three times, unless otherwise noted. ANOVA, Tukey's test and *t*‐test were among the statistical analyses.

## 3 Results

### 3.1 Cell Cytotoxicity and Morphological Analysis

Myristicin's cytotoxic effects on MDA‐MB‐231 cells were assessed using the MTT assay. The fact that the WRL‐68 hepatocellular cells subjected to myristicin (0.1, 0.25, 0.5, 1, 2.5, and 5 mM) for 24 h did not exhibit any notable toxicity and that the cell viability remained above 85% demonstrates that myristicin has low cytotoxicity, as seen in Figure 1b. The anticancer effect of myristicin was also assessed using the MTT test at these same concentrations (Figure 1a). Breast cancer cells exhibited concentration‐dependent cytotoxicity following a 24‐h incubation period with varying myristicin concentrations and the IC_50_ value of myristicin was detected as 0.65 ± 0.98 mM. For every subsequent mechanistic investigation, effective concentrations were chosen.

**Figure 1:**
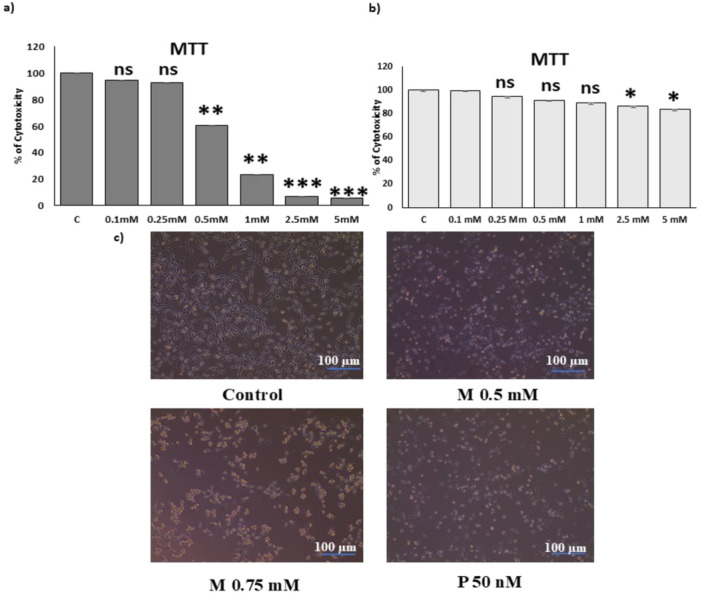
Cytotoxicity potential and Morphological analysis of Myristicin on MDAMB‐231 cells (a) In vitro cytotoxicity effect of various concentrations of Myristicin in TNBC cells and (b) In WRL‐68 normal hepatocyte cells after 24 h incubation was evaluated by MTT. *Denotes statistically significant differences in comparison with control *p < 0.05, **p < 0.01, ***p < 0.001 when compared with control cells. (c) Representative Phase‐contrast images display the morphological changes under the treatment of Myristicin. Magnification, ×10. Scale bar = 100μm.

Apoptosis‐related cellular transformations include cell rounding, membrane rupture, cell compression, cell death body development, and more. Using phase‐contrast microscopy, we have seen these changes in myristicin‐treated cells. Myristicin caused well‐known morphological changes that were comparable to untreated control cells, according to the results presented in Figure 1c.

### 3.2 Apoptosis Induction by Myristicin

In order to determine whether myristicin's growth inhibition was connected to apoptosis, myristicin treated triple‐negative breast cancer cells were examined under a fluorescent microscope. When cells were treated with varying myristicin concentrations using Hoechst stain, the number of apoptotic cells increased in a dose‐dependent manner, as seen in figure 2a. The cellular alterations of myristicin‐treated cells were further investigated using AO/EtBr dual staining. According to the dual staining, myristicin‐treated cells had chromosomal condensation and ultra‐microstructural alterations, which gave them an orange to red color, whereas the control cells showed an intact nuclear structure with green fluorescence (Figure 2b). In comparison to untreated cells, the primary development of apoptotic bodies was observed in treated groups.

**Figure 2:**
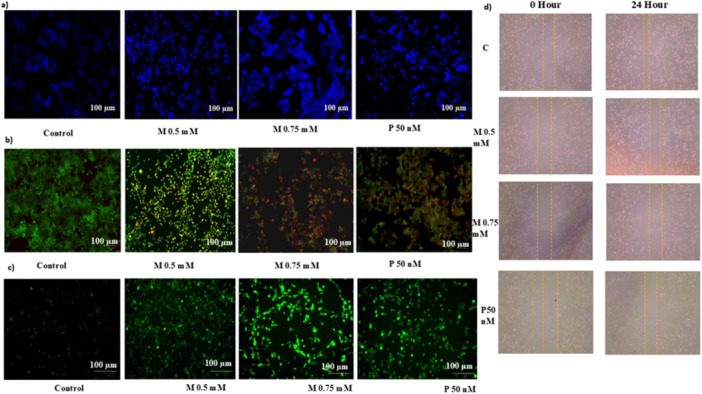
Myristicin induced apoptosis mechanism in MDAMB‐231 TNBC cells. (a) Hoechst‐stained fluorescent images showing the nuclear fragmentation of TNBC cells treated with Myristicin and Paclitaxel. (b) Representative image showed the Live and Dead cells by acridine orange/ethidium bromide dual staining with myristicin treatment after 8 h. (c) Representative images of ROS staining with ‘,7’ dichlorofluorescein diacetate (DCFDA) dye in MDAMB‐231. Magnification, ×40. Scale bar = 100 µm. (d) A phase contrast image of the scratch assay effect of myristicin and paclitaxel for 24 h incubation. Magnification, ×4. Scale bar = 200 µm.

### 3.3 Measurement of ROS Generation Within Cells

An important factor in controlling apoptosis is a surge in ROS production that occurs when the mitochondrial membrane potential changes. We used 2’,7’‐dichlorofluorescein diacetate (DCFDA) staining in fluorescent microscopic analysis to determine the accumulation of ROS within the cells. The findings displayed in Figure 2c showed that ROS increased in a concentration‐dependent manner. Myristicin increased the amount of green fluorescence in the cells when compared to cells that were not treated. According to the findings, myristicin considerably raised intracellular ROS in cancer cells, which may have caused ROS‐mediated DNA damage or been linked to the induction of cell death.

### 3.4 Effect of Myristicin on Apoptosis by Annexin V/PI

The annexin V‐FITC/PI flow cytometer was used to investigate the apoptosis of MDAMB‐231 cells treated with myristicin (0.5 mM and 0.75 mM). The flow cytometry‐detected graph is displayed in Figure 3a. The percentage of early cell apoptosis of myristicin‐treated cells was dose‐dependently enhanced compared with the control group, reaching 46.4 ± 2.31% at a higher concentration of myristicin.

**Figure 3:**
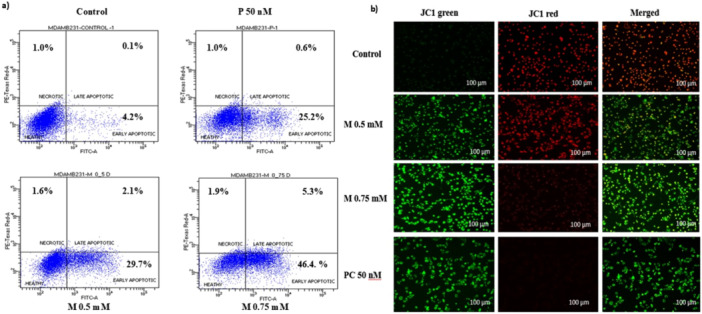
The apoptosis induction and membrane potential of Myristicin on MDAMB‐231 cells. (a) Representative flow cytometry histograms illustrating apoptosis stimulation in TNBC cells stained with Annexin V/PI. (b) Representative fluorescent images of JC‐1 staining of MDA‐MB‐231 cells after treatment with myristicin.

### 3.5 Evaluation of Mitochondrial Membrane Potential

Mitochondria are important in the regulation of apoptosis, and the disruption of the mitochondrial membrane potential is considered an early marker of the apoptotic process. In the present study, the effect of myristicin on mitochondrial membrane potential was evaluated by fluorescent probe JC‐1. The intensity of red fluorescence was decreased, and the intensity of green fluorescence was increased during depolarization of the mitochondrial membrane. Mitochondrial membrane potential loss was observed with myristicin treatment in a concentration‐dependent manner, indicating a significant mitochondrial depolarization with increasing concentrations of the compound, as shown in Figure 3b.

### 3.6 Determination of Cell Migration

Myristicin's antimigratory properties were examined using the scratch/wound assay, which measures the amount of wound closure in a 24‐h period in an artificial wound made in the cell monolayer. In figure 2d, phase‐contrast images demonstrated that, while migration peaked in control cells over a 24‐h period, myristicin treatment significantly decreased the migratory potential of cancer cells while maintaining a similar wound area.

### 3.7 Investigation of In Vivo Anticancer Efficacy

The anti‐tumor activity of myristicin was further assessed using 4T1 tumor‐bearing Balb/c mice, which was prompted by the in vitro inhibitory activity of myristicin. Figure 7f indicates the tumor volume for the various groups of tumor‐bearing mice in the course of the study, with normal saline serving as a control. According to the findings, myristicin hindered tumor growth more than the breast cancer control. Figure 7d illustrates the mouse body weight curve over time. Furthermore, the relatively constant average weight of the mice in each group indicates that myristicin lacks general toxicity. The tumor progression of the mice in each group at the final stage of the study is represented in Figure 7b,c. Compared to the untreated control, the mean tumor weight of myristicin has declined (*p* < 0.01). In addition, the survival rate was considerably increased by the myristicin group compared with the breast cancer control group in Figure 7e. H&E staining was used to demonstrate the treatment impact from a cellular perspective. It revealed that the tumor tissues of the myristicin‐treated group had an elevated level of cell apoptosis with nuclear rupture and nuclear‐cytoplasmic atrophy, further suggesting that myristicin may successfully hinder tumor growth (Figure 7g). Additionally, we analyzed the serum antioxidant enzymes (origin kit) and DHE staining of the tumor using a fluorescent microscope. From the findings, we observed that myristicin enhanced the antioxidant enzymes such as catalase, SOD, and GST, and increased ROS generation in the tumor. The findings of these experiments are consistent with the potential apoptosis mechanism.

### 3.8 Measurement of Antioxidant Enzymes

Oxidative stress is primarily facilitated by the malfunctioning of naturally occurring antioxidant enzymes such as catalase, superoxide dismutase, GST, and others. The enzyme assay kit (origin) was used to measure the levels of the most prominent enzymatic antioxidant enzymes in serum, including catalase, superoxide dismutase, and GST. In comparison to the untreated breast cancer group, the myristicin‐treated group markedly enhanced the enzyme levels of catalase and superoxide dismutase and lowered the activity of the GST enzyme (Figure 13a‐c).

### 3.9 Activity of LDH Enzyme and MDA

We observed the LDH activity in the mice serum, we observed that myristicin treatment significantly reduced the LDH activity, as shown in Figure 8a. Furthermore, since malonaldehyde (MDA) is a byproduct of lipid peroxidation and serves as a biological marker of oxidative damage throughout BC, its activity in serum was investigated. Based on the results, we proved that the group treated with myristicin reduced their MDA concentration in comparison to groups that were not treated (Figure 8b).

### 3.10 DHE for Superoxide Production Detection

Superoxide generation was detected by using the oxidation products of DHE (Dihydroethidium), which produced orange‐fluorescent fluorophores. These products have the ability to attach to DNA as well, and since we used the blue‐fluorescing DAPI to counterstain nuclei. Based on the results, we observed that the intensity of fluorescence of DHE is greater than DAPI fluorescence, resulting in a red nuclear appearance in the myristicin‐treated group. These findings confirm the higher superoxide production in the myristicin group compared to the untreated breast cancer group (Figure 8c).

### 3.11 Evaluation of Gene Expression Associated With Apoptosis

The molecular mechanism of myristicin‐induced apoptosis in cells and tissues was investigated by measuring the expression levels of several apoptosis‐related genes. The SYBR Green‐based quantitative real‐time PCR (Bio‐Rad) was used for evaluating the relative gene expression of Caspase 8, Bax, Bid, PARP, Bcl2, p53, Caspase 9, Caspase 3, p21, NF‐kB, TLR2, FADD, Survivin and Cdk1. In both cells and tissues treated with myristicin, the expression level of pro‐apoptotic genes was significantly greater than that of untreated breast cancer control. Moreover, myristicin‐treated groups showed downregulated expression of anti‐apoptotic genes (Figure 4a–k & Figure 9a–f).

**Figure 4:**
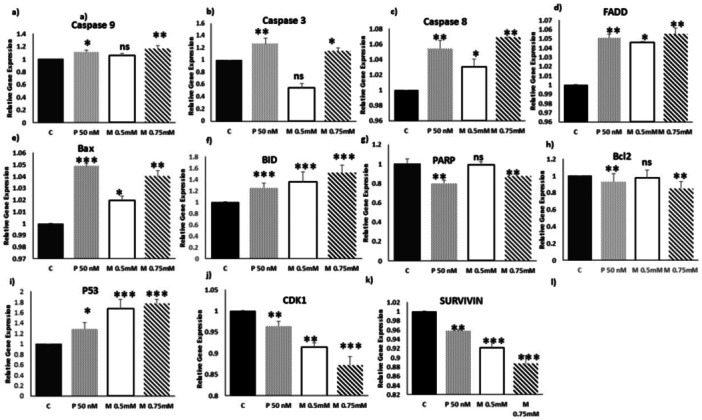
(a−l) Disclosure of relative gene expression level of Caspase 9, caspase 3, caspase 8, Bax, BID, PARP, Bcl2, p53, Cdk1 and Survivin in MDAMB‐231cells after 24 h treatment with different concentrations of Myristicin. *Denotes statistically significant differences in comparison with control *p < 0.05, **p < 0.01, ***p < 0.001 when compared with control cells.

### 3.12 Role of Myristicin in Mitochondrial Mediated Pathways and Apoptosis

The Western blot analysis revealed that certain proteins are involved in apoptotic pathways. The results, as illustrated in Figure 5a–e and Figure 6a,b,e, showed that the pro‐apoptotic proteins, including cleaved PARP, caspase 9, caspase 3, caspase 7, cleaved caspase 9, p53, and Bad, exhibited elevated expression levels in myristicin‐treated cells. Besides, myristicin also down‐regulated the antiapoptotic proteins expressed in Figure 6f,g. The identical β‐actin blot served as the loading control in Figure 5f and 6e. Compared to the untreated in vivo breast cancer group, the histogram (Figure 10a–g and 11a–e) revealed that the protein expression level of the myristicin‐treated group was significantly up‐regulated for the pro‐apoptotic and down‐regulated the anti‐apoptotic key proteins. Furthermore, we evaluated the significance of SDHA, HSP60, and cytochrome C proteins involved in mitochondrial stress pathways and observed potential regulation in groups treated with myristicin. These altered protein characteristics may stimulate the apoptotic pathway in BC cells and tumors. The distinct β‐actin blot was used as the loading control in Figure 10g and 11e.

**Figure 5:**
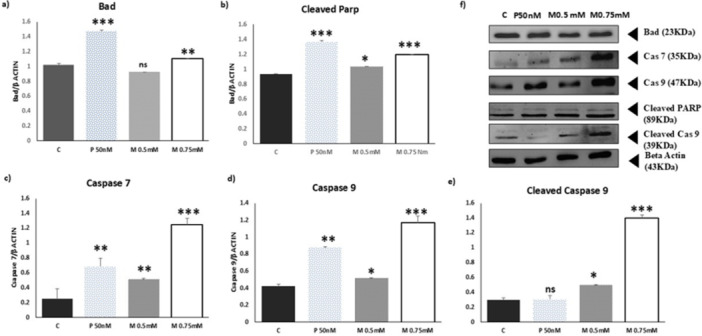
(a−e) Bar diagrams of western blot revealing the relative intensity band for c‐PARP, Bad, Caspase 7, Caspase 9, and c‐Caspase 9 in MDAMB‐231 cells treated with Myristicin and Paclitaxel for 24 h. (e) Western blot analysis showing the upregulation of proteins. Beta‐actin was used as the internal control. Results are expressed as the mean ± SD (*n* = 3) *p < 0.05, **p < 0.01, ***p < 0.001 when compared with control cells.

**Figure 6:**
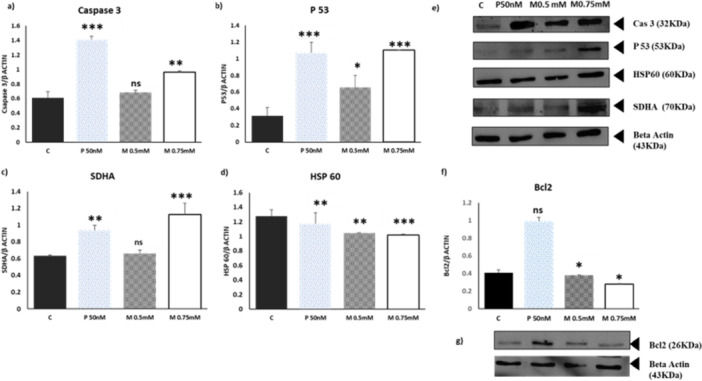
(a−d) Bar diagrams of western blot showing the relative intensity band for caspase 3, p53, SDHA, and HSP60 in MDAMB‐231 cells treated with various concentrations of Myristicin and Paclitaxel for 24 h. (e) Western blot analysis showing the upregulation of caspase 3, p53, and SDHA proteins and downregulation of HSP60. (f, g) Illustrate the bar diagram and relative intensity band of Bcl2 protein. Beta‐actin was used as the internal control. Results are expressed as the mean ± SD (*n* = 3) *p < 0.05, **p < 0.01, ***p < 0.001 when compared with control cells.

**Figure 7:**
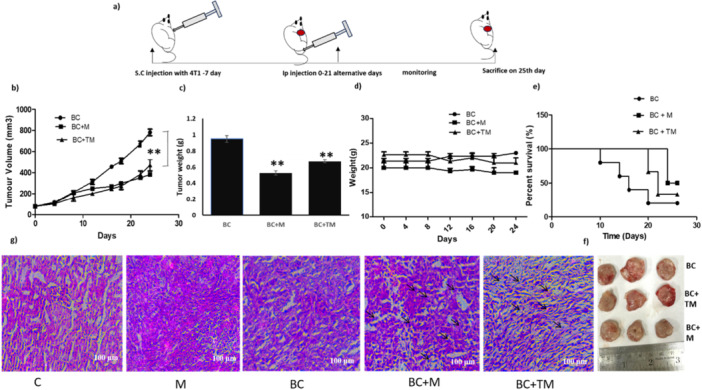
(a) Schematic representation of the experimental design for Tumor induced Balb/c mice model by 4T1 cells. (b) Evaluation of tumor volume; (c) Assessment of tumor weight (d) measurement of body weight. (e) Kaplan–Meier survival rate of mice (f) images of tumor collected from mice on day 25 (g) H&E staining sections of normal mammary gland tissues from the healthy control (C) and myristicin‐treated (M) groups, and tumor tissues from the 4T1‐induced breast cancer groups (*n* = 6). Scale bar 100 µm. *p < 0.05, **p < 0.01 as compared to breast cancer control.

**Figure 8:**
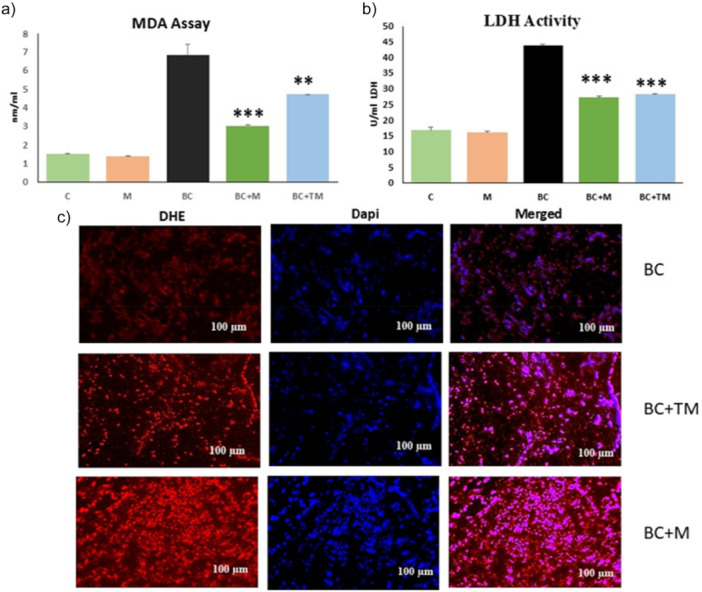
(a, b) Represents a graph showing the release of LDH and MDA of mice serum (n = 6). *Denotes statistically significant differences in comparison with breast cancer control, *p* < 0.05, **p < 0.01, ***p < 0.001. (c) Fluorescent images showing the DHE staining of tumor tissue. Scale bar 100 µm.

**Figure 9:**
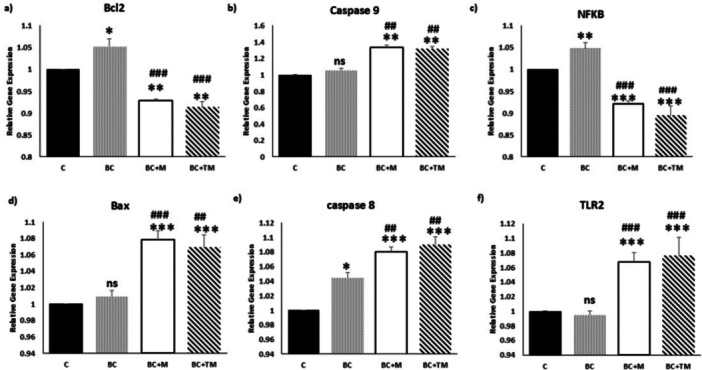
(a−f) Detection of relative gene expression of Caspase 9, caspase 8, Bax, Bcl2, NFkB and TLR2 in myristicin‐treated tumor (*n* = 6). Data are representing the mean ± SD (*n* = 3) *p < 0.05, **p < 0.01, ***p < 0.001 when compared with normal control. ^#^ *p* < 0.05, ^##^ *p* < 0.01, ^###^ *p* < 0.001 when compared with non‐treated breast cancer control.

**Figure 10:**
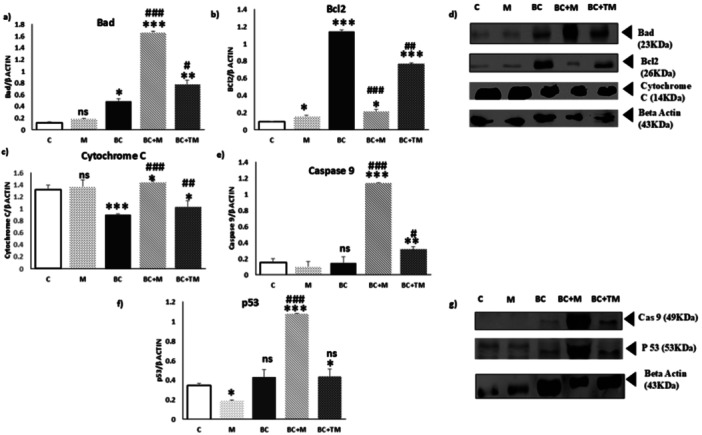
**(**a–g) Bar diagrams and relative intensity bands of western blot showing the expression of apoptotic proteins including Caspase‐9, Bcl2, Bad, p53 and cytochrome C in tumors treated with myristicin (*n* = 6). *Denotes statistically significant differences in comparison with control: ns > 0.05, *p < 0.05, **p < 0.01, ***p < 0.001. #Denotes statistically significant in comparison with breast cancer control group: #*p* < 0.05, ##*p* < 0.01, ###*p* < 0.001. ^#^ *p* < 0.05, ^##^ *p* < 0.01, ^###^ *p* < 0.001.

**Figure 11:**
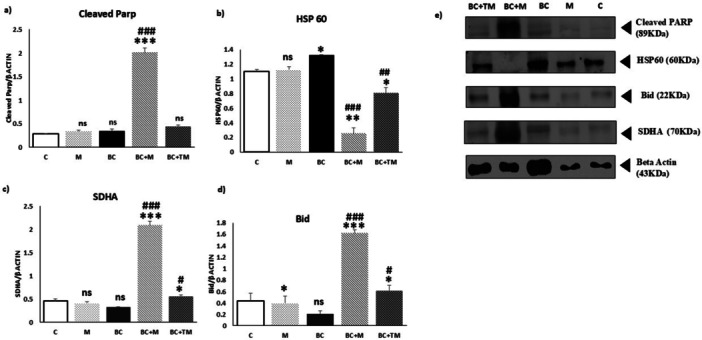
(a−d) Bar diagrams of western blot revealing the relative intensity band for c‐PARP, Bid, SDHA, and HSP60. (e) Western blot band showing the upregulation and down‐regulation of proteins involved in the apoptosis. Beta‐actin was used as the internal control. Results are expressed as the mean ± SD (*n* = 3) *p < 0.05, **p < 0.01, ***p < 0.001 when compared with normal control. ^#^ *p* < 0.05, ^##^ *p* < 0.01, ^###^ *p* < 0.001 when compared with breast cancer control.

### 3.13 Safety Profiles

At the conclusion of the antitumor study, the tumors in the myristicin treatment group were fixed and H&E stained in order to further evaluate the toxicity and potential applications of myristicin. No abnormalities were produced by any of the groups in the kidney, liver, spleen, lung, or heart, according to the H&E results (Figure 12a). Blood samples were taken for serum biochemical analysis following 23 days of treatment in healthy Balb/c mice. The results showed that all parameters, including SGOT, Creatine Kinase, ALP, and Urea, were within the normal range (Figure 13d–g). The results mentioned previously verify myristicin's outstanding biocompatibility, lack of adverse reactions, and inability to adversely affect liver and kidney function when applied to the long‐term treatment of breast cancer.

**Figure 12:**
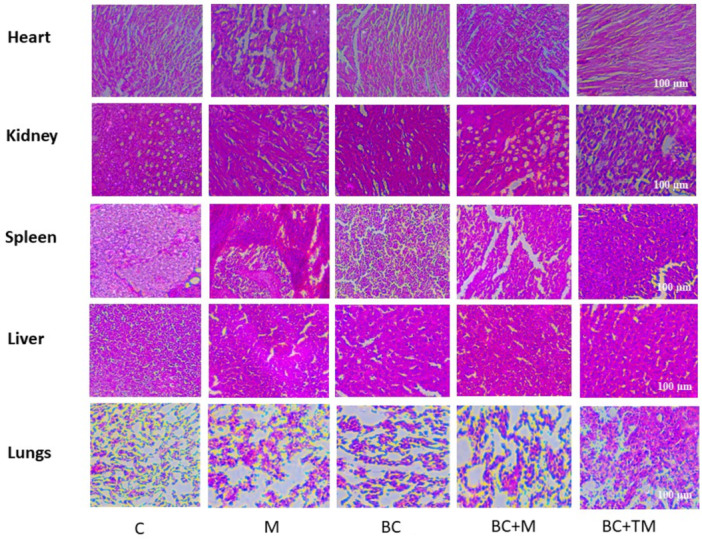
In vivo toxicity measurement. Pathological sections of major organs obtained from normal control, myristicin control, BC control, BC + M, and BC + TM treated mice were examined by H&E staining after the end of the treatment (*n* = 6). Scale bar, 100 μm.

**Figure 13:**
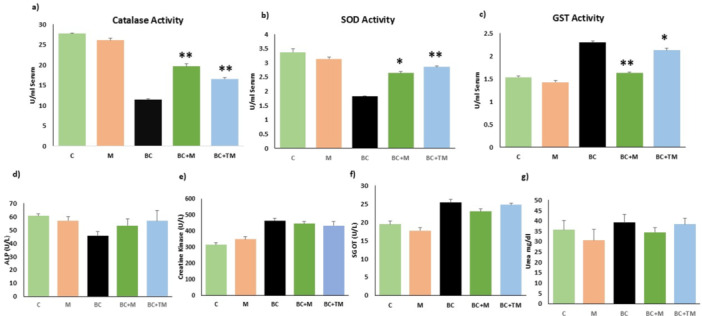
(a–c) Represent the antioxidant enzymes activity of serum (*n* = 6). Catalase (a), SOD (b), GST (C). (d–g) Toxicity measurements: ALP (d), CK (e), SGOT (f), Urea (g) of different treatments.

## 4 Discussion

Many factors, including a poor prognosis, limited options for therapy, an elevated mortality rate, and more, make TNBC a challenging disorder to manage. TNBC, an aggressive form of breast cancer that is often associated with a greater prevalence of gene mutations and negative ER, PR, and HER2 receptors, is difficult to cure in clinical trials because it lacks therapeutic targets and is resistant to chemoradiotherapy, despite the availability of various cancer therapy techniques [22]. The predominant therapy for TNBC is still cytotoxic chemotherapy because hormonal treatments are ineffective in this situation. In TNBC, typical chemotherapy regimens include medications that target DNA repair complexes such as taxanes and cisplatin, compounds that target p53, compounds that target cell proliferation, and specific targeted therapy (mTOR, FGFR, VEGF, and EGFR inhibitors) [23]. Administering a natural compound as a component of therapy is a desirable alternative to using traditional medications for treatment in order to optimize findings.

The current study investigated the effect of the natural compound “myristicin” in TNBC treatment. Some natural products, particularly those obtained from medicinal herbs, have a strong regulatory role in different diseases [24, 25]. The potentially active ingredient in Myristica fragrans (nutmeg), myristicin, has a variety of therapeutic uses, such as anti‐inflammatory, anti‐bacterial, anti‐fungal, antioxidant, anti‐diabetic, and analgesic properties. Furthermore, nutmeg plants have been utilized extensively to treat inflammation, flatulence, stomach pain, and stimulants [26]. Certain studies have demonstrated that myristicin inhibits the proliferation of several kinds of cancer cells; significant benefits have been observed in hepatocarcinoma [27] and other solid tumor cells, including human oral cancer cells [28]. Nowadays, there is extensive research focused on discovering natural substances that stimulate apoptosis in cancerous cells. Furthermore, limited is understood about the molecular processes and cellular morphological alterations that underlie myristicin‐induced apoptosis in triple‐negative breast cells.

According to our study, acridine orange/ethidium bromide and Hoechst‐stained fluorescent microscopy revealed nuclear shrinkage, blebbing, and developed extracellular and/or intracellular apoptotic bodies. In addition, apoptosis was measured using flow cytometry to identify phosphatidylserine surface expression. When the myristicin concentration was higher, the 24‐h apoptotic indexes of MDA‐MB‐231 cells, which was evaluated using Annexin V/PI staining, were 46.4%. Statistically significant variations were observed when compared to the control group. While breast cancer cells in the control group underwent impulsive apoptosis, the apoptotic index was small. As a result, we believed myristicin might trigger the death of breast cancer cells and ultimately inhibit the growth of tumors.

The signaling cascade in apoptosis focuses on two distinct events: mitochondria‐dependent and mitochondria‐independent pathways. Any damage or destruction of DNA, or an increase in the production of free radicals, triggers this mitochondria‐dependent pathway. In contrast with healthy cells, tumor cells produce more free radicals [29, 30, 31]. In addition to balancing their cell developmental processes and proliferation, tumor cells exhibit boosted intracellular ROS during tumorigenesis [32]. On the other hand, high generation of ROS increases cell instability and stimulates apoptosis signaling pathways in malignant cells [33, 34]. The DCFH‐DA staining assay was used in the present investigation to determine intracellular ROS using a fluorescent image. The findings demonstrated that, following an 8‐h incubation period, myristicin dose‐dependently raised the generation of ROS in MDAMB‐231 cells. ROS‐induced ROS release, or a higher level of ROS release from damaged cells, is the name used to describe this kind of system [35]. Antioxidant enzymes defend against oxidative damage and prevent excessive ROS production [36]. Research showed that the functions of antioxidant enzymes are decreased in the progression of a subset of breast cancer cases [37]. Following compound treatment of tissues, we measured the activity of the antioxidant enzymes catalase, SOD, and GST. The findings indicated that, in comparison to the untreated group, the activity of catalase and SOD was increased and the GST enzyme level was reduced.

Lactate is present in significant amounts in malignant cells, and recent investigations have shown a clinical association between tumor levels of lactate and increased metastasis, recurrence, and inadequate response to therapy [38]. Lactate dehydrogenase, which can be found by LDH serum profiling, leaks into the bloodstream when a cell is damaged. Serum LDH levels can be very important as a marker of prognosis for patients with a variety of solid cancers, including colorectal cancer [39], lung cancer [40], nasopharyngeal cancer [41], prostate cancer [42], and breast cancer [43]. In addition, malondialdehyde is an indicator of oxidative stress, particularly in a number of medical conditions associated with the lipid peroxidation process [44]. Increased oxidative stress and undermined antioxidant defenses are indicated by a marked rise in MDA and a decline in antioxidants in cancers. This event has a significant impact on the onset of disease and tumor development brought on by gene mutations brought on by elevated MDA levels [45]. Our findings showed that in the myristicin‐treated group, the serum values of MDA and LDH achieved nearly normal levels.

An essential component of tissue homeostasis, intrinsic or mitochondrial apoptosis is a mechanism of apoptosis that has been conserved throughout evolution. The B cell lymphoma 2 (BCL‐2) protein family, whose several members collaborate and oppose one another to ultimately determine the strength of the mitochondrial outer membrane (MOM), is primarily responsible for controlling the pathway [46]. In the cytoplasm and mitochondria, HSP60, a 60 kDa molecular chaperonin mainly referred to as Cpn60/HSPD1, plays a crucial and passive role in the folding of unfolded proteins and simple polypeptides [47]. Distinct cancers demonstrate HSP60 in various ways. Most cancers, including colorectal, breast, hepatocellular, and oral cancers, as well as non‐small‐cell lung cancer (NSCLC), overexpress HSP60 [48]. On the other hand, when HSP60 expression is reduced in healthy cells, the level of p53 rises, triggering p53‐dependent apoptosis and death of cells. The tumor microenvironment and tissue type affect HSP60 expression. HSP60 inhibits the release of cytochrome C from mitochondria and ceases apoptosis in a number of tumors by binding to numerous molecules in the cytosol, including Bak and Bax [49]. One of the mitochondrial enzymes, succinate dehydrogenase (SDH), also called mitochondrial Complex II, has a unique capacity to take part in the electron transport chain and the citric acid cycle, where it oxidizes succinate to fumarate. As for the SDH complex's diagnostic efficiency in medical situations, research has shown that both its subunits and its metabolite succinate can function as biomarkers across a variety of tumors [50, 51]. In this study, we showed that, in comparison to untreated groups, myristicin treatment significantly upregulated SDH and downregulated Hsp60 expression levels in BC cells and tissue.

Mitochondria are important regulators of the intrinsic apoptotic pathway, and the collapse of mitochondrial membrane potential (ΔΨm) is considered to be one of the earliest events that triggers programmed cell death [52]. In the present study, ΔΨm loss was visualized by JC‐1 staining and was found to be dependent on the myristicin concentration, as the red fluorescence decreased and the green fluorescence increased, which indicates mitochondrial depolarization. The collapse of ΔΨm is coupled to mitochondrial outer membrane permeabilization, leading to the release of cytochrome c and subsequent activation of caspase‐9 and downstream executioner caspases, resulting in apoptosis. Mitochondrial dysfunction has been shown by recent studies to be a key event linking oxidative stress to apoptotic signaling in cancer cells, and loss of ΔΨm is a reliable marker of mitochondrial‐mediated apoptosis [53]. Thus, the observed reduction in mitochondrial membrane potential along with our observations of enhanced ROS generation and activation of apoptotic proteins suggest that myristicin induces apoptosis through ROS‐mediated mitochondrial dysfunction and activation of the intrinsic apoptotic pathway.

The present study showed that myristicin triggers MDA‐MB 231 cells to undergo apoptosis by regulating mitochondrial enzymes. A transcriptional factor called the tumor suppressor p53 promotes apoptosis in part by triggering the expression of Bax [54, 55]. P53 has the ability to modify intracellular ROS levels, is crucial for cell death, and can regulate the cell cycle mechanism. Furthermore, p53 may be linked to the upregulation of caspase proteins in both intrinsic and extrinsic apoptotic pathways, and it triggers cancer cell apoptosis by cell cycle arrest at G1 or interphase [56]. Our research demonstrated that myristicin‐treated cells and tissue also exhibited regulation of p53, Bax, Bcl2, and mitochondrial proteins. Inhibiting proapoptotic counterparts is one way that the mitochondrial apoptosis pathway inhibitor Bcl‐2 is employed [57, 58]. In this investigation, myristicin‐treated cells showed a significant reduction in Bcl‐2 expression, whereas the expression of mitochondrial stress proteins, including Cytochrome C and SDHA, was significantly increased and HSP 60 was down‐regulated. In addition, elevated levels of Bax, Bad, and caspase 9, 3, and 7 led to an increase in the Bax/Bcl‐2 ratio. Cytochrome c subsequently activates Caspase‐9 and the downstream effector caspases, including Caspase‐3 and Caspase‐7, leading to PARP1 cleavage and cell death through apoptosis [59, 60]. The present study emphasizes the increased levels of Cytochrome c observed in whole‐tissue lysates, alongside the changes in Bcl‐2 family proteins and the activation of Caspase‐9, Caspase‐7, Caspase‐3, and cleaved PARP1, highlighting the significance of the intrinsic mitochondrial apoptotic pathway. However, given that Cytochrome c was assessed using whole‐tissue lysates rather than distinct cytosolic and mitochondrial fractions, these findings ought to be interpreted as suggestive of mitochondrial participation rather than conclusive evidence of Cytochrome c dispersing into the cytosol. Real‐time PCR outcomes also demonstrated that myristicin‐treated cells and tissues expressed upregulation of proapoptotic genes (caspase 9, caspase 8, caspase 3, Bid) and downregulation of antiapoptotic genes (Bcl‐2, P21, cdk1, parp) compared to untreated cells and tissues.

Our earlier research showed that myristicin reduced cell viability and triggered apoptosis in estrogen receptor‐positive MCF‐7 breast cancer cells by influencing apoptotic signaling pathways via key markers such as cleaved PARP, Cytochrome c, and Caspase‐9 [20]. While the current study offers a more detailed mechanistic analysis in TNBC by investigating regulators of apoptosis, encompassing the Bcl‐2/Bid pathway, and confirming these molecular results in an *i*n vivo breast tumor model. In contrast to MCF‐7 cells, MDA‐MB‐231 cells do not express any receptors, which renders them unresponsive to endocrine and HER2‐targeted therapies, thereby enhancing their aggressive phenotype, elevated metastatic capability, and unfavorable clinical prognosis [2]. The efficacy of myristicin on both ER‐positive and TNBC cells indicates that its anticancer properties might engage shared downstream apoptotic pathways that are not reliant on hormone receptor signaling. The originality of this study lies in the incorporation of an in vivo breast tumor model, offering physiological validation for the in vitro results. The current study greatly broadens our earlier results by showing, for the first time, that myristicin shows marked antitumor effects against TNBC in both in vitro and in vivo models by activating the intrinsic apoptotic pathway.

The current study demonstrated that myristicin triggers programmed cell death in human triple‐negative breast cancer cells by upregulating proapoptotic proteins and downregulating antiapoptotic proteins. We subsequently examined myristicin's effectiveness in Balb/c mice injected with a 4T1 tumor‐bearing model. In mice treated with myristicin, tumor volume was reduced, and there was no obvious liver, spleen, heart, or kidney damage. The antitumor efficacy of myristicin was comparable with tamoxifen in in vivo experimental parameters. Significantly, myristicin demonstrated greater anticancer efficacy than tamoxifen across various experimental measures. Furthermore, the minimal toxicity observed in this study implies that myristicin could be explored as an adjunct therapy to conventional chemotherapy medications. Combination therapy can enhance treatment efficacy while allowing for reduced doses of cytotoxic drugs, which aids in minimizing side effects, delays the emergence of drug resistance, and improves patient outcomes. However, additional research on pharmacokinetics, pharmacodynamics, and combination efficacy, as well as comprehensive toxicological evaluations in appropriate animal models, is required before myristicin's therapeutic potential can be utilized in clinical settings. Given the potential and safety of myristicin, it is a promising candidate for the therapy of breast cancer, although additional studies are needed.

## 5 Conclusion

Here, we proposed a natural compound called “myristicin” for the application in triple‐negative breast cancer in both in vitro and in vivo. In vitro investigation, we found that myristicin initiated dose‐dependent mitochondrial‐mediated apoptosis, which was followed by an increase in Bax/Bcl‐2 ratios and led to apoptosis. In MDA‐MB‐231 cells, myristicin triggered apoptosis, antimigration, and cell cycle arrest and also regulated mitochondrial proteins. The 4T1 xenografted BALB/c mice model was used for further investigation of the potential role of myristicin in apoptosis pathways, and encouraging findings were noted. The mice's survival rate rose, and the tumor volume was dramatically reduced. According to this strategy, myristicin may have been developed into a low‐toxicity, highly effective treatment for breast cancer.

## Author Contributions

Conceptualization: Janeesh Plakkal Ayyappanand and Sudhina Sufina Nazar. Literature collection: Sudhina Sufina Nazar. Data curation and original draft writing: Sudhina Sufina Nazar. Reviewing, editing, and finalizing the manuscript: Sudhina Sufina Nazar, Aswathy Vadakkumkattle Ajaykumar and Janeesh Plakkal Ayyappan. All authors have read and agreed to the final version of the manuscript.

## Ethics Statement

All ethical guidelines were followed with the committee for the purpose of control and supervision of experiments on animals (CPCSEA), and ethical sanction no. IAEC 2‐KU‐6/2022‐BCH‐JN (7) for the conduct of animal experiments. This study was conducted in compliance with the ARRIVE guidelines.

## Conflicts of Interest

The authors declare no conflicts of interest.

## Data Availability

The data that support the findings of this study are available from the corresponding author upon reasonable request.
